# ‘Before, we ended up in conflicts, now we can provide support’—Experiences of Community Reinforcement and Family Training (CRAFT) for parents of young adults with hazardous substance use

**DOI:** 10.1186/s12888-024-05913-x

**Published:** 2024-06-21

**Authors:** Ola Siljeholm, Joachim Eckerström, Olof Molander, Jennie Sundbye, Anders Hammarberg

**Affiliations:** 1 Centre for Psychiatry Research, Department of Clinical Neuroscience, Karolinska Institutet, & Stockholm Health Care Services, Region Stockholm, Stockholm, Sweden; 2 Stockholm Centre for Dependency Disorders, Stockholm Health Care Services, Region Stockholm, Stockholm, Sweden

**Keywords:** Community reinforcement and family training, Thematic analysis, Cognitive behavioral treatment, Qualitative research, Concerned significant other, Hazardous substance use, Young adults, Parental support program

## Abstract

**Background:**

The prevalence of hazardous substance use is highest in the age between 18 and 25, but few young adults enter treatment. Community Reinforcement and Family Training (CRAFT) is a support program for concerned significant others (CSOs) of individuals with diverse substance use disorders and is proven efficacious in promoting treatment entry. The aim of the current study was to investigate the experiences of CRAFT among parents of substance using young adults.

**Methods:**

We used a qualitative design conducting semi-structured interviews with 10 parents of young adults (18–24 years) with hazardous substance use. The participants were recruited from a randomized controlled trial of the CRAFT program. The transcribed interviews were analyzed using thematic analysis.

**Results:**

We divided the results into three overall domains—Reasons for entering the CRAFT program, Strengths of the CRAFT program and Limitations of the CRAFT program – with three to four themes under each domain. The parents appreciated the accessible support at a time when they needed it due to feelings of shock and powerlessness, and they described communication strategies together with positive reinforcement as the two most helpful CRAFT-sessions. Regarding limitations of CRAFT in the current population, the parents wanted more accessible support for the young adults when they were ready to enter treatment, and described difficulties to practice CRAFT-components due to changing life-circumstances and fear of aggravated health for their young adults.

**Conclusion:**

The results provide arguments for the health care system to implement support programs to parents of young adults with hazardous substance use. The results show that CRAFT is suitable for the current population, but with some possible additions due to changing circumstances that are common in the young adult developmental phase emerging adulthood.

**Trial registration:**

The trial was pre-registered at isrctn.com, reference number ISRCTN12212515 date: November 7, 2018.

**Supplementary Information:**

The online version contains supplementary material available at 10.1186/s12888-024-05913-x.

## Background

The prevalence of alcohol and drug use is highest in the age between 18 and 25 [1–3], for some leading to hazardous use with increased risks for negative physiological, psychological, and social consequences. For some individuals, the consequences are so extensive that they meet the criteria for substance use disorder (SUD) [4, 5]. However, young adults with hazardous substance use or SUD (for the sake of convenience henceforth referred to as hazardous substance use) are rarely motivated to seek help, with only approximately 10% entering treatment [6–8]. Parental support and/or pressure, and the importance of the relationship to the parents, is among the most common reasons for seeking treatment mentioned by young adults [9–11]. After turning 18 years old, it is common for young adults in Sweden, as well as in many other countries, to terminate treatment that was initiated by their parents if the young adults do not perceive themselves in need of help [1], and parents also loose the possibility to participate in treatment unless invited by the young adults [12, 13]. For parents of young adults who do not engage in treatment, no structured support is offered within the regular health care system in most countries [6].

The prevalence of psychiatric and somatic conditions among concerned significant others (CSOs) to individuals with substance use problems is higher than in the general population [14–17]. Parents of young adults with hazardous substance use is argued to represent an even more burdened group with elevated strains due to the parent–child bond [13, 18–20]. In qualitative studies, parents describe emotional experiences of powerlessness, grief, shame, guilt, stress and stigma, financial issues and social consequences like being blamed by others for causing the substance use [18, 20–25]. Parents also emphasize stress caused by the struggle between potentially enabling substance use, for example by allowing their young adult children to stay at home although having used substances, or by paying off debts, versus letting the young adults handle all potential negative consequences of the substance use [18]. Hence, support programs directed at parents of young adults with hazardous substance use are warranted both as an aid to handle the emotional, financial and social distress perceived [26, 27], and also as a possible means to promote treatment entry or reducing substance use in the young adults [6, 28].

### Community reinforcement and family training (CRAFT)

Community Reinforcement and Family Training (CRAFT) [29], is a manualized support program with the aim of supporting CSOs to motivate a substance using relative to enter treatment, while simultaneously increasing CSOs’ quality of life, and is based on cognitive behavioral therapy (CBT) and motivational interviewing (MI). In CRAFT, CSOs are taught new strategies with three main goals [30]: 1) to improve their own quality of life; 2) to support the relative’s sober and healthy activities, thereby hopefully decreasing the substance use, and; 3) to promote help seeking behavior in the relative. The efficacy of CRAFT has been investigated in populations with different problematic substances and CSOs with different relationships to the using individual, with treatment entry rates varying between ~ 20 to 86% [29, 31–36]. Further, CRAFT has been shown to improve mental health and relationship functioning for the participating CSOs and resulted in a reduction in substance use [32, 37, 38]. Two non-randomized studies have investigated CRAFT for parents of substance using adolescents/youths [39, 40], both reporting treatment entry rates between 60–70%. Neither study included interviews with the participating parents, which is of importance when evaluating the administration of an intervention to a new target population.

Three previous qualitative studies examining the experiences of participants in CRAFT-programs, although performed in different contexts regarding for example delivery modes, program content, settings, and participants, have been identified [41–43]. Hellum et al. interviewed 11 female CSOs of individuals with alcohol use disorder (AUD) after participating in a trial of CRAFT delivered within the Danish Addiction Service [41]. Osilla et al. conducted a feasibility trial where CRAFT was administered using a web-based design to spouses of military veterans concerned about their partners alcohol consumption, and interviews were performed with eight participants [42]. Finally, Siljeholm & Ekström interviewed 13 female mothers sharing a child with a co-parent with unhealthy alcohol use after taking part in a web-based self-delivered program comprising components of CRAFT and parental management training (PMT) [43]. Both Hellum et al. and Osilla et al. found that the positive communication component of CRAFT was the most helpful, followed by reinforcement of positive behaviors [41, 42]. Hellum et al. also concluded that CRAFT was perceived by the CSOs as helpful in improving quality of life regardless of modality and that CSOs were helped by a better understanding of AUD [41]. Siljeholm & Ekström found that the mothers reported an improved relationship to their children, increased own positive activities and less adaptation to the co-parent as main effects of the CRAFT + PMT program [43].

The present qualitative study is a sub-study of a randomized controlled trial (RCT) where the efficacy of CRAFT compared to manualized counselling was investigated for parents of treatment refusing young adults (18–24 years old) with hazardous substance use. Results from the RCT have been published in [44], but a short description follows bellow.

### Short description of the RCT

Participants in the RCT were recruited via advertisement in social media and through two outpatient clinics for young adults in Stockholm, Sweden. Initially, recruitment was from the wider Stockholm area, but due to the COVID-19 pandemic, delivery mode was changed to videoconference, and recruitment could be performed nationwide. After a screening process, a total of 113 participants were randomized (ratio 1:1) to either CRAFT (*n* = 58) or manualized counseling (*n* = 55). Both interventions were conducted through individual sessions involving the participant (and co-parent if relevant). For the initial 52 participants, all sessions were delivered face-to-face, while for the remaining 61 participants, sessions were delivered via videoconference.

In accordance with previous CRAFT-trials, the main outcome in the RCT was treatment engagement among the young adults, with secondary outcomes including young adults’ substance use and relationship happiness. Parents who succeeded in motivating their young adults to seek treatment were given suggestions by the therapists as to where such treatment could be provided. Due to that the study was carried out within the general health care system, a procedure for immediate access to treatment for the young adults was not provided, a procedure which in prior studies has shown to increase treatment entry rates [33].

At the 6 months follow-up, 33% of CRAFT-participants and 31% of counselling participants had reported young adult treatment entry, with no difference between conditions. Participants reported clinically relevant reductions in young adult alcohol and substance use and increased relational happiness, with no differences between conditions [44]. Further no difference in any outcome was shown between participants who received the interventions face-to-face vs via videoconference.

To our knowledge, no previous study has adapted a qualitative approach to investigate the experiences of CRAFT among parents of substance using young adults, which was the overall aim of the current study. More specifically, the aim was to explore the participants’ descriptions of why they entered the CRAFT program, which CRAFT components that were considered the most and least helpful and if the participants perceived that CRAFT was able to help them to reduce negative effects stemming from the young adults’ substance use on themselves and their young adults. The study is important in order to complement the findings from the aforementioned RCT, with the aim to adapt CRAFT to the target population prior to implementation in the clinics.

## Methods

### Design

In this qualitative study, we conducted interviews with parents who had participated in CRAFT via videoconference in the RCT described in [44]. The Consolidated Criteria for Reporting Qualitative Research (COREQ) [45], was utilized to foster transparency of the qualitative research process, see Appendix 1.

### CRAFT

CRAFT in the RCT comprised of eight individual sessions á 45–60 min with themes in accordance with the CRAFT manual [30], adapted for parents of young adults through appropriate case descriptions and facts about relevant substances. See Table 1 for an overview of program content.

**Table 1:** Summary of CRAFT sessions content^a^

**1. Introduction and motivational enhancement:** Problem description and expectations Previous strategies to handle young adult’s substance use Reinforcers and motivations for change Preventing and coping with violent behavior **Exercise:** State desired goals by CRAFT participation
**2. Functional analysis of young adult’s substance use:** Analysis of situations Triggers and consequences **Exercise:** Functional analysis
**3. Positive communication skills:** Components of positive communication and role-playing exercise **Exercise:** Practice communication skills
**4. Encouraging sobriety and positive reinforcement:** Signs of substance use Positive reinforcement of sobriety **Exercise:** Create a plan for positive reinforcement
**5. Parent’s well-being:** Rational for own well-being Goals to enhance well-being **Exercise:** Follow through on a goal to enhance well-being
**6. Managing young adult’s substance use:** Analyses of responses Unintentional enabling Time-out from positive reinforcement, retraction of rewards Natural negative consequences **Exercise:** Handling young adult’s substance use
**7. Problem-solving and treatment engagement:** Model for problem-solving Windows of invitation to treatment Suitable treatment options Role-playing exercise in treatment encouragement **Exercise:** Invite young adult to treatment
**8. Summary and maintenance of results:** Summarize program content Identify progress Maintenance of positive changes

^**a**^Apart from the first session, the inherent order of sessions could be altered based on the therapist’s judgement of the parent’s needs

The CRAFT intervention was administered by a team consisting of a social worker, a psychiatric nurse, and a clinical psychologist. The therapists had undergone a comprehensive three-day training in CRAFT and had prior experience of working with the method both in individual and group format.

### Control condition

The control condition was a manualized form of counselling which comprised five individual 45 min sessions and one group psychoeducation session. Session’s themes were: 1) Problem description, parent’s concern and understanding young adult’s substance use; 2) Parents response to young adult substance use; 3) Mapping of young adult’s social networks; 4) Relational patterns in the family, and 5) Follow-up session one month after the fourth session. The group psychoeducative session included information about drugs and effects on the developing brain and a group discussion with other parents.

### Participants

The main eligibility criteria for participation in the RCT were: i) identified substance use in the young adult; ii) young adult currently refusing to enter treatment; iii) parent and young adult in contact at least 40% of the days and iv) parent not showing problematic alcohol- or substance use. For further eligibility criteria, see [44]. For the current qualitative study, purposive sampling was used, and we approached parents via telephone following completion of the 24-week follow-up assessment which was also the primary endpoint of the RCT. In total, 15 participants were approached, one participant did not answer, four participants declined due to perceived lack of time, and one did not show up on the appointed time. We performed nine interviews with a total of ten informants since one co-parent, who had been a part of all CRAFT sessions, also participated in the interview. Characteristics of the interviewed parents are presented in Table 2.

**Table 2:** Demographics of interviewed parents and their young adults compared with non-interviewed CRAFT participants in the RCT [44]

	*Interview sample n* = *10*	*CRAFT sample in RCT n* = *49*
**Parent**		
Gender, female, *n* (%)	9 (90.0%)	45 (91.8%)
Age parent (years), mean (*SD*), range	50.9 (5.45), 41–61	51.0 (5.35), 38–61
Country of birth, Sweden, *n* (%)	8 (82.5%)	40 (81.6%)
Education (College), *n* (%)	8 (88.9%)	30 (61.2%)
**Young adult**		
Gender, male, *n* (%)	8 (88.9%)	40 (81.6%)
Age (years), mean (*SD*), range	20.0 (1.87), 18–23	19.8 (1.87), 16–23
Entered treatment during RCT	2 (22.2%)	17 (34.7%)
Days with substance use last month of RCT, mean (*SD*), range	2.3 (2.25), 0–5	7.2 (9.1), 0–24
Days with alcohol use last month of RCT, mean (*SD*), range	5.1 (3.7), 0–10	3.9 (4.0), 0–16
*Main problematic substance, n (%)*		
Cannabis	4 (44.4%)	28 (57.1%)
Alcohol	1 (11.1%)	5 (10.2%)
Other (Cocaine, opioid analgesics, amphetamines, benzodiazepines)	4 (44.4%)	16 (32.7%)

### Data collection

All interviews were conducted by authors OS or JS between September 2021—May 2022 via videoconference or by telephone. The interviews lasted between 30–60 min and only the researchers and interviewees were present during the interviews. OS was involved in the RCT-study in all parts from study design to statistical analysis, JS was study coordinator and had hence met most of the participants in the screening phase six months prior to the interviews. All interviewees were aware of the roles of the interviewers and that a future goal of the trial was to implement the CRAFT intervention in regular practice, but the participants were encouraged to be frank about their experiences since such an approach was of importance to provide valid information in evaluating program content and future adaptations for clinical practice. The interviews followed a semi-structured interview guide (Appendix 2), with open-ended questions and suggestions for prompts and examples. The participants were also asked specifically which CRAFT sessions they had found most and least helpful. The interviews were recorded and subsequently transcribed verbatim by a professional transcriber. Transcripts were not returned to the interviewees for commenting, but the interviewees were informed that they could contact the interviewers if they wanted to add anything. No pre-defined criteria of data saturation were employed during the data collection.

### Data analysis

A thematic analysis was performed on the complete data set using the method described by Braun and Clarke [46]. The purpose was to summarize and interpret the data content in relation to the study aim using an inductive approach. The analysis comprised six phases: 1) Data familiarization, 2) Generating codes, 3) Searching for themes, 4) Reviewing themes, 5) Defining and naming themes, and 6) Producing the report. This iterative process was conducted via both physical and digital meetings. In phase 1, the authors reviewed the transcripts to gain a comprehensive understanding of the data corpus. The close involvement in the RCT of author OS came with a risk of bias, so to manage prior knowledge or pre-understanding of CRAFT, the data analysis was carried out by authors JE and OM, who had not been involved in the project previously. In phase 2, JE and OM extracted all smaller units, such as phrases or sentences, which were relevant for the study aim. To ensure coding reliability and reduce the risk of researcher subjectivity, JE and OM coded all smaller units together. During phase 3, all codes were analyzed, and initial themes were derived from the data. In phase 4, all authors met together to review the initial themes and compare analysis in a triangulation process. As the next step, JE and OM continued with phase 5 and processed the definition and naming of the themes. In the last phase, JE and OM finalized the results, and OS, JE, OM, and AH were involved in the writing of the manuscript with JS providing feedback. All analyses were performed in Microsoft Excel and Microsoft Word. See Table 3 for examples of smaller units in relation to codes and themes.

**Table 3:** Examples from the thematic analysis

Smaller units	Codes	Themes	Domains
“Because it was a shock when, when I found out.” (Informant no. 3)	Feeling of shock	Feeling of powerlessness	Reasons for entering the CRAFT program
“And we felt that we ended up in a lot of admonishing, he just stood there and took it or it turned into conflict and quarrel, and we felt that it didn't lead to anything. And with what we got in session three and four, it gave us completely different tools. (Informant no. 2)	Conflicts, got communication tools	Acquired communication skills	Strengths of the CRAFT program
”That there is some follow-up after 3 and 6 months, together with the person you had the program with, to see how we succeed in maintaining these tools that we have learned.” (Informant no. 2)	Suggests follow-up	Lacked follow-up for parents	Limitations of the CRAFT program

### Ethical considerations

This study followed the ethical standards of the World Medical Association Declaration of Helsinki [47]. The research project was approved by the Swedish Ethical Review Authority (region of Stockholm) (Diary No: 2021–04538). We informed all participants about the study both via written material in advance and in conversation before engaging in the interview and collected oral informed consent.

## Results

The results were divided into three overall domains that corresponded to our three areas of interest: *Reasons for entering the CRAFT program*, *Strengths of the CRAFT program* and *Limitations of the CRAFT program*. The thematic analysis of the interviews with parents who have experience of the CRAFT program is presented under each domain, see Table 4.

**Table 4:** Overview of the results

Domains	**Reasons for entering the CRAFT program**	**Strengths of the CRAFT program**	**Limitations of the CRAFT program**
*Themes*	***Feeling of powerlessness*** ↓ ***Easily accessible support*** ↓ ***Gain increased knowledge and strategies to help young adults***	***Acquired communication skills*** ↓ ***Shifted focus from substance use to encouragement*** ↓ ***Improved relationship*** ↓ ***Helped young adults to reduce substance use and seek treatment***	***Difficulties to practice CRAFT components***
			***Lacked follow-up for parents***
			***Challenges when trying to affect substance use***
			***Insufficient support and treatment options for young adults***

The arrows illustrate that there is a process between themes, meaning that the previous theme created conditions for, or led to, the following theme

### Reasons for entering the CRAFT program

The analysis of the parents' narratives resulted in the following themes regarding reasons for entering the CRAFT program: *Feeling of powerlessness*, *Easily accessible support* and *Gain increased knowledge and strategies to help young adults.*

### Feeling of powerlessness

The time from parents becoming aware of their young adults’ substance use up to entering CRAFT differed substantially within the sample. One parent was more or less still in shock when starting CRAFT since she only a few weeks earlier had discovered that her daughter had used cocaine, while other parents reported cannabis and extensive use of alcohol since about five years back. Some parents reported early discoveries, often regarding cannabis, among their young adults, while other parents had started to suspect problems by noticing changes in the young adults’ behavior, such as skipping school and staying out late at night. When confronted, the young adults often admitted to having used substances and said they would stop, but over time, more incidents occurred, which made parents feel that they lacked power to change the situation:

*“He has repeatedly promised to stop* [using cannabis] *and so on, but he doesn’t, and then there has been a number of, well, incidents when he has used and come home under the influence and so on. And we felt a little powerless in that, I guess.*

(Informant no. 2)

For some parents, the young adult’s substance use had intensified over several years, leading to progressively worse incidents:

*For a while there it was pitch black. The situation derailed, he became very aggressive and violent and broke things at home, such as windowpanes. He backfired and became a completely different person when he drank.”*

(Informant no. 8)

Several parents felt that they had no control over the situation or ability to help their young adults, which created a mix of emotions, including frustration, powerlessness, and a feeling of shock and not knowing what to do. This led parents to seek information and help.

### Easily accessible support

The parents described that they needed all the help they could get. They searched for information on the internet, made phone calls to different agencies, and found it hard to access support for parents when their young adults weren't involved and didn’t want to participate. The parents reported that most support services assume that the young adults take the initiative to seek help, before the parents can get involved. Several parents explained that they encountered the CRAFT program by coincidence, such as through advertisement or when it was mentioned in a nationwide radio broadcast:

*“We were worried about him, and we didn't really know what to do (…). And then by chance I was sitting in the car on the way home from work and heard in a radio program about this study. And then I looked it up and we were lucky enough to participate.”*

(Informant no. 7)

The parents were grateful that some support was available for them at all. They found the CRAFT program to be accessible and a quick path to help. Parents expressed appreciation that the support program was held digitally, which facilitated their participation as they did not have to take time off from work and, in some cases, both parents could be involved.

### Gain increased knowledge and strategies to help young adults

Several parents applied to the CRAFT program since they perceived the young adults’ substance use as a serious and challenging phenomenon, which they lacked information or knowledge about. Parents also reported that they wanted to be able to communicate better in order to “reach” their young adults and wanted advice on how to approach or relate to their young adults’ problems.

Some parents described that they wanted to influence their young adults, e.g., to “get free from their problem behavior to be able to live a normal life” and wanted information about available help for their young adults to seek. At the same time, parents also acknowledged the complexity in a situation where they were trying to seek help for someone that didn’t want help. One parent described conflicting motifs; from one perspective seeking tools to change her young adult, but from another point of view not changing him too much. The interviewed couple made the following comment:

*“- Actually, it’s a reflection of the whole system. It’s not that obvious what to do when you want to help someone close to you, how to do that in this system.*

*- When, well, when the person doesn’t want any help. Or can’t receive any help, perhaps one should say.”*

(Informants no. 9 & 10)

### Strengths of the CRAFT program

**Fig. 1:**
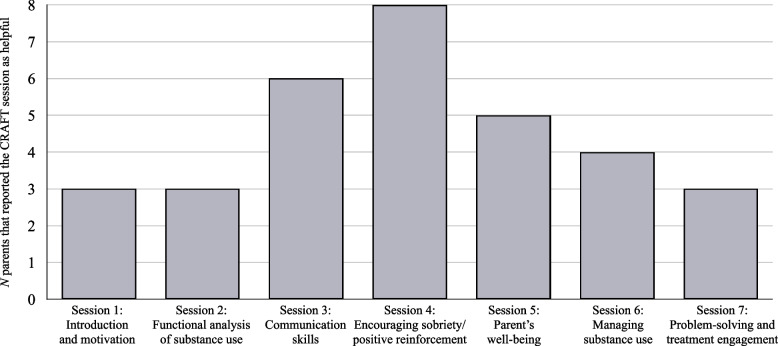
The most helpful CRAFT sessions (multiple answers possible), as reported by interviewees (*N* = 10)

All parents reported that they would recommend the CRAFT program to others facing similar challenges. Although it requires effort from the parents to complete homework, they found it to be helpful and beneficial, and one parent even described participation in the study as life-changing. Several strengths of the treatment were identified in the thematic analysis, resulting in the following themes: *Acquired communication skills, Shifted focus from substance use to encouragement, Improved relationship* and *Helped young adults to reduce substance use and seek treatment*. The parents’ collected answers on most helpful sessions are showed in Fig. 1.

### Acquired communication skills

The interviews showed that the CRAFT program trained the parents in altering their communication towards their young adults. Many parents reported having no previous strategy on how to approach their young adults, leading to long arguments and emotional conflicts, mostly about substance use. Understanding the benefits of being less confrontational, more clear, direct, and concise in their communication, was described by parents as extra valuable. The parents found it helpful to choose the right moment for conversation by assessing, as one parent stated, “whether the traffic light is green or red”. Instead of arguing and fighting in situations that previously had caused such negative interactions, they tried to let the situation be and awaited a better time for dialogue. This felt counterintuitive, but it was beneficial to direct more energy towards what the parents wanted to see more of. The learned communication skills felt supportive even long after the program ended.

*“Training in changing the way I communicate with my son is probably the biggest change. I got to learn strategies for how I should think about it.”*

(Informant no. 1)

### Shifted focus from substance use to encouragement

Parents described an increased understanding on why people use substances, which was helpful as a means of empathy towards their young adults and to see the importance of support to overcome substance use. Parents reported that this increased knowledge about substance use, together with the newly learned communications skills, helped them to shift the subject of conversation. Instead of feeling stuck and only focusing on the substance use, they tried to highlight and support what was positive in life and to find such activities to perform together. Hence, the CRAFT-component *Encouraging sobriety and positive reinforcement* was reported as helpful by many parents as an integral part in shifting focus towards constructive aspects of young adults’ behaviors.

*“In some way, I realized that addressing it when he's down won't get you anywhere. Instead, the key to success is trying to make him think that life is fun.”* (Informant no. 9)

Parents described that the shift from negative aspects to positive also led to changed attitudes between the parents and the young adults. Fewer conflicts resulted in less guilt for the parents and it felt like a beginning to create a more sustainable family life. The parents still expressed their thoughts about substance use clearly, but in a less confrontative manner than before, and they tried to be clear that they disliked the behaviors but loved their young adults.

### Improved relationship

Despite the challenges posed by the young adults’ substance use, the parents felt that their relationship with their young adults had improved after implementing the communication skills they had learned. Some parents described how the relationship had changed over time, and that the relationship was very good before the substance use, and then rapidly deteriorated due to conflicts. But recently, after the program, it had started to improve again. One parent reflected about how the improved relationship affected the substance use:

*“I believe that he values our opinion and well-being, not only for our sake but also for his own. He doesn't want to cause us distress and this has likely played a role in his decision not to use drugs. He also acknowledged that drug use is not working for him.”* (Informant no. 6)

Other factors, such as the young adults growing older and maturing, and external circumstances like changing social circles, were also described as having a positive impact on the relationship. Parents described their relationship as more open and honest after the program. They tried to make it clear that they prefer to hear the truth, whether they like it or not. For some parents, the improved relationship made it more evident that their son or daughter needed professional help to stop substance use.

### Helped young adults to reduce substance use and seek treatment

Several parents reported that their participation in the CRAFT program, and the improved relationship that it led to, was a contributing factor for their young adults to become open to receive treatment, or to reevaluate their views on substance use. Parents believed that their own altered behavior, in terms of new communication skills and shifted focus to encouragement, had a positive impact. It was easier to approach their young adults in the challenges they were facing and to be supportive. Before CRAFT, the young adults completely refused to go to treatment, but now there was an opening, which one parent describe in following words:

*“So that he has taken a first step in actually saying that he now wants to go and talk to someone.”*

*(Informant no. 2)*

### Limitations of the CRAFT program

During the interviews the parents described issues in relation to their involvement in the CRAFT program, which were themed into four potential areas of limitations: *Difficulties to practice CRAFT components, Lacked follow-up for parents,*

*Challenges when trying to affect substance use,* and *Insufficient support and treatment options for young adults*.

### Difficulties to practice CRAFT components

Some parents reported difficulties practicing CRAFT strategies due to specific life-circumstances at the time of engaging in the support program. First, several parents reported that it was hard for them to find time to increase their own life-quality, as they felt they needed to prioritize their young adults’ situation, and/or felt a constant worry for them. For instance, one parent described that:

*“I felt I didn’t have time to… well, take care of myself. I wanted to focus on him first, at the time (...). For instance, if my husband and I would go out to dinner, we shouldn’t talk about our problems, but sort of like, talk about other things (...). I guess it was good that we did that, but it felt hard for me to prioritize.”*

(Informant no. 6)

Second, parents reported that it was hard for them to let their young adults face the natural consequences of their substance use. Parents described that this part of the CRAFT program was important, but that they struggled with it, e.g., because they did not want the consequences to be too severe, that their young adults would “sink so deep”. One parent reported that:

*“The problem was that when we had that dialogue* [about facing natural consequences]*, it was during the hardest period, and I feared that he would kill himself (...). So, for me it wasn't relevant to, so to speak, freeze him out if he had behaved badly (...). Um… Because I somehow just wanted to make him see the meaning of life.”.*

(Informant no. 8)

Third, several parents reported that their young adults moved out of the home during the time of the study, naturally leading to less time spent together. The distance created by this change in young adults’ residence deprived the parents of many opportunities to practice certain components such as positive reinforcement and face natural consequences of substance use. To some parents however, mainly those describing a less severe substance use in their young adults, the distance was perceived as somewhat beneficial, as it helped them to realize that their children had turned into adults that could handle their own issues.

### Lacked follow-up for parents

Most parents expressed the need for additional support after the CRAFT program had ended. The most common request was for more follow-up visits with the CRAFT therapist (and possibly including the young adults), e.g., at three or six months, or by scheduling appointments on demand. Parents described that such meetings could be important to sustain the CRAFT strategies they had acquired in the program, to receive continuous support, or just to be able to talk to somebody about how to handle situations they were struggling with.

### Challenges when trying to affect substance use

Some parents reported that their young adults’ substance use did not change because of their participation in the CRAFT program. A few parents had tried to talk to their young adults about seeking help, but it had failed as the young adults did not have the same perspective on their substance use. Other parents said that they had not come as far in the process yet to be able to speak to their young adults about their substance use.

Parents described that it was hard for them to be sure about the frequency of substance use as it occurred outside of their home or “under the radar”, which caused worry to some parents. However, to other parents, letting go of controlling behaviors was a conscious strategy. When asked about her young adult’s current substance use, one parent responded:

*“I can’t really give an answer to that (...). I know there is substance use. But to investigate it further, you do the things you shouldn’t do, by using control strategies. And those I have let go.”*

(Informant no. 1)

Another reason that several parents mentioned regarding unchanged substance use was the importance of the young adults’ social contexts as reinforcers of substance using behavior. One parent described how her son lost contact with most of his positive milieus when the Covid-19 pandemic started and instead engaged with “bad company” that introduced him to substance use. Another parent explained how her daughter was not susceptible to arguments that her substance use was hazardous:

*“She says that it’s like that, that everybody drinks. That’s her standard answer, everyone uses drugs (…) she’s no different from the others in that respect. She even uses less drugs and drinks less alcohol than many others, she says”*

(Informant no. 3)

In some cases where a decrease in substance use had occurred, parents described that it was triggered by social consequences in relation to friends and relationships outside of the family. For example, one parent recounted that her son had an “eyeopener” when he was thrown out of the apartment he was sharing with a friend due to his substance use, causing him to move back home, and his cannabis use reportedly decreased significantly as a result. Another parent said that her son’s drinking behavior had changed markedly, albeit not yet entirely “cured”, after the destructive romantic relationship he was in had ended. A third parent had just found out that her son was not allowed to rent an apartment from a friend’s relative since the relative was aware of his recurring substance use. The parent saw that this had affected her son and was now planning to use this information as motivation to change.

### Insufficient support and treatment options for young adults

Several parents raised an issue of insufficient support and treatment options for the young adults after the CRAFT program had ended and expressed concerns that the program had lacked “a bridge” to easily accessible support. Several parents suggested that the young adults could be included in CRAFT-sessions, or that there would be a previously determined treatment provider ready to make a scheduled appointment for their young adults. Parents described that the lack of treatment options led to stress and frustration as they were “left alone to find help”. They portrayed the path towards a more open dialogue about substance use and discussing treatment seeking as fragile, and that it is crucial that support is available when the young adults are motivated. One parent described that she had to make quite an effort to find a “way in” to treatment. When her son finally called the addiction center, he was told that he could not schedule an appointment until three months later. This parent emphasized that:

*“What I want to convey to the study is the importance of having an entrance into healthcare that is easily accessible. When you have come so far it is important that there are no remaining barriers. That it’s possible to schedule an appointment with a clinician available who can meet the youth, and not being told ‘we don’t have time for you right now’”.*

(Informant no. 4)

## Discussion

In this study, we interviewed 10 parents of young adults with hazardous substance use after the parents had participated in the support program CRAFT. To our knowledge, this is the first study to investigate parents’ experiences of a CRAFT program by adapting a qualitative approach. The main findings were that the parents greatly appreciated the possibility of accessible support at a time when they needed it and that communication strategies and positive reinforcement were identified as the two most helpful sessions. Regarding limitations of CRAFT in the current population, main results included parents lacking easily accessible support for the young adults when they were ready to enter treatment, and difficulties to practice CRAFT-components due to changing life-circumstances and fear of aggravated young adults’ health.

CRAFT was offered via the RCT [44], and was available to the parents for a limited time period. The parents’ accounts of how crucial it was to receive support when needed is a main finding in the study and a strong argument for the implementation of support programs for parents into regular health care. Through skills learnt in CRAFT, the parents in our study describe how they succeeded in improving the relationship to their young adults, which in some cases led to the young adults reevaluating their substance use and, in some cases, enter treatment. The importance of easily accessible support for CSOs has been described in previous research, for example as means to reduce stigma, to validate and empower the CSOs, to improve the quality of life to the CSOs and to possibly improve the relatives’ substance use [22, 43, 48, 49]. A tentative conclusion is hence that offering easily accessible support for parents could possibly result in a decreased risk for young adults to develop SUD and other negative consequences, although longitudinal data would be required to establish long term outcomes of the CRAFT intervention. A similar conclusion is presented by Waldron et al. after delivering CRAFT to parents of substance using adolescents [39], which led to treatment engagement for 67% of the adolescents. The authors suggest that CRAFT could be a cost-effective alternative to more extensive methods for treatment engagement such as juvenile justice interventions [39].

Another aspect regarding easily accessible support is the digital delivery mode with sessions via videoconference, which was appreciated by almost all interviewed parents. Only one parent explicitly stated that she would have gained more from receiving CRAFT face-to-face due to difficulties concentrating when in front of a screen. Further, some parents mentioned that the quality of an interaction face-to-face usually was somewhat better than via video, but all parents expressed that they would still recommend other parents to seek the intervention. Since only parents who received CRAFT via video were interviewed, we cannot compare the experiences to parents who received the intervention face-to-face. However, there were no differences in either participant characteristics or treatment outcomes between parents who received CRAFT via video or face-to-face [44]. Hence, the positive aspects of providing CRAFT via videoconference, such as availability, is inferred to greatly outweigh any potential negative aspects such as difficulties to concentrate or a marginally better interaction quality.

Although being helped by CRAFT, a vast majority of the parents expressed that they lacked easily accessible treatment options for their young adults. Several parents stated that they didn’t know where to turn or that their young adults were not offered an appointment promptly. Providing easy access to treatment when the relative is motivated is stressed by the founders of CRAFT as an important part of the intervention [30], and facilitating treatment entry for the substance using relatives, either with the same team of therapists providing CRAFT or linked to another prespecified institution, has been effective in many previous CRAFT trials [31, 33, 50]. As previously mentioned, it was not possible to provide an integrated treatment option procedure in the study, due to that the interventions were delivered within the general health care system, not allowing a fast track into treatment ahead of other waiting patients. Hence, it is plausible that the treatment engagement rate in the RCT [44], might have been higher if there would have been an option for integrated treatment for the young adults. However, in the context of Swedish public health care, we consider the current CRAFT-trial as having high ecological validity, showing that CRAFT provide positive outcomes both for parents and young adults, regardless not being able to provide an integrated treatment option. Nevertheless, based on our results, we propose that CRAFT for parents should enable an integrated treatment option for the young adults in order to increase treatment entry rates, and we recommend that such an option is established when possible.

We found that the parents’ main reasons for seeking support were related to feelings of shock after discovering that their young adults used substances and that parents felt powerless following unsuccessful attempts to decrease the frequency of substance use, together with a wish to gain strategies to affect their young adults. Similar themes of shock and powerlessness have been described in several other studies on parents of substance using grown adults (e.g. [19, 21, 51]). Hence, we infer that the powerlessness-theme is connected to the parents’ stated desire to gain knowledge regarding substances, i.e. to obtain new strategies to help their young adults. Many parents also requested knowledge about different substances, for example how they affect the brain or how they are produced. In CRAFT as conducted in the current study, some information was provided, but mainly limited to signs on how to detect if the young adult was under the influence (to enable reinforcement of sober behaviors and time-out from positive reinforcement). It is hence suggested that a more comprehensive module with psychoeducative material regarding different substances and how they affect the evolving brain is added when administering CRAFT to parents of young adults. Corroborating our results, a wish for increased knowledge and improved strategies for dealing with the relatives’ substance use are themes that have been described as reasons for entering CRAFT in other contexts [41–43].

Regarding the different CRAFT components, the parents in our sample perceived the sessions on communication strategies and positive reinforcement as the most helpful, a conclusion based on both interview data and in parents’ specification of helpful sessions (Fig. 1), corroborating previous findings in qualitative studies of CRAFT regarding most helpful sessions [41, 42]. The communication skills were described as a key strategy that helped them to smoother interactions with their young adults, to improve the relationship between parents and young adults and to provide new possibilities to reinforce positive young adult behaviors. Specifically, it appears to have been successful for the parents to plan how and when to discuss the potential dangers of using different substances (especially cannabis). Debates over substance use are often difficult to “win” since many young adults usually can provide ample arguments from substance positive websites, friends, or other sources. For the parents in our sample, choosing appropriate timing and strategies to discuss substance use was described as particularly helpful. We hence propose that the aspect of timing is highlighted in the positive communications session when delivering CRAFT to parents of young adults. The shifts in parental behaviors from confrontative to more positively oriented, were described as crucial parts for the young adults to become open to change, either through help-seeking or through reduced substance use. Further similarities to previous qualitative studies on CRAFT include the parents’ descriptions of the significance of gained knowledge regarding both the mechanisms of substances, and how substance use can be understood via analyses of triggers and consequences (via the CRAFT component functional analysis) [41, 42]. In essence, our results suggest that corresponding components that have been identified as most relevant to CSOs in other contexts also appeal to parents of substance using young adults.

Some limits of CRAFT in the current population were identified based on the informants’ descriptions. For example, several parents acknowledged challenges characterized by that the most rewarding reinforcers of young adult behaviors came from environments outside of the parent-young adult context. Several parents described how substance use occurred in “bad company” or at parties among friends, and that they could not reach their young adults with arguments even with gained improved communication skills through CRAFT. On the other end of the spectrum, negative consequences in other social contexts (regarding accommodation with friends) caused two young adults to reevaluate their substance use, which made them both more open to discuss negative aspects of substance use with their parents. Changing circumstances and strong social reinforcers outside of the family are natural parts of the developmental phase often referred to as *emerging adulthood.* This phase describe the development of young adults approximately between the age 18–25, is a time of changing parent–child dynamics, and is characterized by a growing autonomy from parents [52]. We infer from our interviews that the young adults are in the middle of this phase, which is a factor that needs to be accounted for when delivering CRAFT to the current population. Specifically, we believe that it is important to acknowledge the strength of reinforcers of young adult behaviors from contexts outside of the family and suggest that therapists and parents in session could investigate the young adults’ social networks to a higher degree in order to identify positive relations to promote. One example of visualizing the young adult’s social network which also includes to what extent different social groups are associated with alcohol-/substance use is Social Identity Mapping in Addiction Recovery (SIM-AR) [53]. The research on SIM-AR and related literature on social identities as pathways into and out of addiction (e.g. [54]), highlight the role of identity in relation to substance use. Identifying oneself as an “active substance user” leads the individual to approach groups expressing a similar identity, while an identity of being “in recovery” is associated with increased company of non-substance using peers. If parents could recognize social groups with whom their young adults are less likely to use substances, for example appreciated friends the young adult used to spend time with more often in the past, the parents could potentially reinforce the young adult to participate in social activities that promote a non-using identity. Hence, this could result in the identification of reinforcers with higher accuracy to promote sober activities, and in the end, treatment seeking in the young adults.

During emerging adulthood, young adults tend to move out of the parents’ homes (and sometimes back in again), which made practicing CRAFT components more difficult for parents. During emerging adulthood, it is common that young adults move out of the parents’ homes, an important change also described by the parents in our sample. This significant change in relationship made practicing CRAFT components more difficult to apply for parents. When the possibilities for face-to-face communication between parent and young adult decreases, digital communication strategies become increasingly important [55]. One possible addition to the CRAFT program could be to apply text message methodology as investigated by Suffoletto et al., where messages were automatically delivered to young adults´ smart phones at occasions when they typically consumed alcohol [56]. The content and delivery of such messages could potentially serve to increase motivation or as reinforcement of sober/healthy behaviors in the young adult and be planned for together between the parent and the therapists in the session on positive communication.

While the young adults experience new possibilities away from home during emerging adulthood, the parents can sense a loss of control and insight in the young adults’ lives, which, although perfectly normal, can be difficult for some parents [1, 57]. This changing parent–child dynamic was mentioned by several of the parents in our study, but mainly as a positive insight that their child had become an adult and that the parents themself could take a step back. It is recommended that CRAFT-therapists are familiar with the concept of emerging adulthood to normalize potentially painful feelings and guide the parents through age adequate young adult behaviors.

Finally, several parents expressed struggling with ambivalence regarding letting their young adults handle all of the consequences stemming from their substance use. Most parents were aware that their behavior in some sense enabled the substance use to continue but made a choice to continue to protect their young adults from certain consequences out of fear that their young adults would “sink too deep” or even commit suicide. Similar ambivalence is described in depth in a study by Nordgren et al. where parents of substance using adult children report on experiences of the most extreme parts of allowing natural consequences—throwing their (adult) children out of the house—after being advised to do so by social services, relatives or self-help groups [18]. For many parents in their study, these actions led to increased feelings of shame and guilt and did not result in positive changes. The authors conclude that professionals and organizations who meet CSOs need to reflect on how, and to whom, the advice to allow for all potential natural consequences should be given [18]. We agree with this conclusion, but on the other hand, several parents in our trial also described that allowing for natural consequences had been a successful part of their strategy to reduce substance use and had also helped them into taking a step back from being a protecting parent of a child to being a less protecting parent of an adult. In essence, we suggest that it is important for CRAFT-therapists, to acknowledge the qualitative difference between a parent–child relationship and, for example, a spousal relationship when advocating for use of severe consequences for substance use in young adults. In the original CRAFT manual, it is emphasized that therapists need to be careful and empathetic when investigating with the CSOs which natural consequences that the relatives could possibly face [30]. We believe that this suggested empathetic approach provides further support that CRAFT is a suitable support program for the current population.

## Limitations

As described in the data analysis section, several measures to enhance *trustworthiness* (credibility, transferability, dependability, and confirmability) [58], of our results were taken. The measures include detailed descriptions of the phenomenon under study, reflexivity of the authors backgrounds and potential biases, transparency of coding-procedures, ample provisions of quotes and finally an interviewed sample that is argued to be adequately representative of the entire sample of the RCT. Nevertheless, this study had some limitations. First, we did not allow for participants to provide feedback on the findings as is suggested in [45], a step in the analysis process argued to increase credibility or to reduce error in the data [59]. However, we propose that the triangulation approach, where several researchers analyzed the data, is sufficient to ensure credibility of the results presented [60].

Second, we did not use a predefined procedure to assess saturation. We did, however, find that code saturation was achieved after the recruitment of nine participants. This is in line with findings by Henning et al. who have studied the saturation process and reported that > 80% of coding can be expected after six interviews [61]. Our interviews generated a substantial amount of data and a rich range of coding, and for the purposes of this study, nine participants were sufficient.

Some potential limitations regarding the interviewed study sample should be mentioned. First, the proportion of young adults who entered treatment during the RCT was lower in the interviewed sample compared to the CRAFT sample in the RCT (22% vs 35%), potentially indicating that the interviews attracted participants who found it more difficult to encourage their young adult to access treatment.

Further, in the interviewed sample, 89% reported a high level of education compared to 61% of the non-interviewed CRAFT sample in the RCT. The proportion of parents who had completed higher education is substantially larger also in comparison with the general population in Sweden (45% in general) [62]. From our data we cannot infer how parents with a lower degree of education experience the CRAFT program and hence this constitutes a limitation in the current study.

Finally, this study only included parents who had participated in a majority of the CRAFT sessions, and hence left out participants who dropped out of the intervention early, leading to a risk of survival bias. However, 90% of the CRAFT-participants in the RCT [44], completed seven or eight out of the eight sessions and there were hence limited options to recruit participants who did not complete the program.

## Conclusion

The results in our trial provide strong arguments for the health care system to implement support interventions to parents of young adults with hazardous alcohol and/or use of illicit substances. The parents found CRAFT to be valuable by providing tools that resulted in an improved relationship to the young adults, in several cases a decrease in substance use, and in some cases that the young adults sought treatment. The results showed that CRAFT is suitable for the current population, but with some possible additions due to changing circumstances as part of the developmental phase emerging adulthood.

### Supplementary Information

Supplementary Material 1.

Supplementary Material 2.

## Data Availability

In order to protect the participants’ identities, the qualitative data analyzed in the current study have not been made publicly available. The transcribed interviews are in Swedish. Translated pseudonymized data are available from the corresponding author on reasonable request.

## Abbreviations

AUD: Alcohol Use Disorder
CBT: Cognitive Behavioral Therapy
CRAFT: Community Reinforcement and Family Training
CSO: Concerned Significant Other
MI: Motivational Interviewing
PMT: Parental Management Training
RCT: Randomized controlled trial
SUD: Substance Use Disorder

## References

[1] Arnett JJ, Zukauskiene R, Sugimura K (2014). The new life stage of emerging adulthood at ages 18–29 years: implications for mental health. Lancet Psychiatry.

[2] Grant BF, Saha TD, Ruan WJ, Goldstein RB, Chou SP, Jung J, Zhang H, Smith SM, Pickering RP, Huang B (2016). Epidemiology of DSM-5 drug use disorder: results from the national epidemiologic survey on alcohol and related conditions-III. JAMA Psychiat.

[3] Arnett JJ (2005). The developmental context of substance use in emerging adulthood. Journal of Drug Issues.

[4] Lim SS, Vos T, Flaxman AD, Danaei G, Shibuya K, Adair-Rohani H, AlMazroa MA, Amann M, Anderson HR, Andrews KG (2012). A comparative risk assessment of burden of disease and injury attributable to 67 risk factors and risk factor clusters in 21 regions, 1990–2010: a systematic analysis for the Global Burden of Disease Study 2010. The lancet.

[5] Patel V, Flisher AJ, Hetrick S, McGorry P (2007). Mental health of young people: a global public-health challenge. Lancet.

[6] Kirby KC, Versek B, Kerwin ME, Meyers K, Benishek LA, Bresani E, Washio Y, Arria A, Meyers RJ (2015). Developing Community Reinforcement and Family Training (CRAFT) for parents of treatment-resistant adolescents. J Child Adolesc Subst Abuse.

[7] SAHMSA.: Substance Abuse and Mental Health Services Administration (2020). Key substance use and mental health indicators in the United States: Results from the 2019 National Survey on Drug Use and Health (HHS Publication No. PEP20–07–01–001, NSDUH Series H-55).

[8] Ozechowski TJ, Waldron HB (2010). Assertive outreach strategies for narrowing the adolescent substance abuse treatment gap: implications for research, practice, and policy. J Behav Health Serv Res.

[9] Cleverley K, Grenville M, Henderson J (2018). Youths perceived parental influence on substance use changes and motivation to seek treatment. J Behav Health Serv Res.

[10] Cornelius T, Earnshaw VA, Menino D, Bogart LM, Levy S (2017). Treatment motivation among caregivers and adolescents with substance use disorders. J Subst Abuse Treat.

[11] Wagner V, Bertrand K, Flores-Aranda J, Acier D, Brunelle N, Landry M, Brochu S (2017). Initiation of addiction treatment and access to services: young adults’ accounts of their help-seeking experiences. Qual Health Res.

[12] Lindgren E, Söderberg S, Skär L (2016). Being a parent to a young adult with mental illness in transition to adulthood. Issues Ment Health Nurs.

[13] Tambling RR, Russell B, D’Aniello C (2022). Where is the family in young adult substance use treatment? The case for systemic family therapy for young adults with substance use disorders. Int J Ment Heal Addict.

[14] Ray GT, Mertens JR, Weisner C (2009). Family members of people with alcohol or drug dependence: health problems and medical cost compared to family members of people with diabetes and asthma. Addiction.

[15] Bischof G, Bischof A, Velleman R, Orford J, Kuhnert R, Allen J, Borgward S, Rumpf HJ (2022). Addiction. Prevalence and self-rated health and depression of family members affected by addictive disorders: results of a nation-wide cross-sectional study.

[16] Casswell S, You RQ, Huckle T (2011). Alcohol's harm to others: reduced wellbeing and health status for those with heavy drinkers in their lives. Addiction.

[17] Di Sarno M, De Candia V, Rancati F, Madeddu F, Calati R, Di Pierro R (2021). Mental and physical health in family members of substance users: a scoping review. Drug Alcohol Depend.

[18] Nordgren J, Richert T, Svensson B, Johnson B (2020). Say no and close the door? Codependency troubles among parents of adult children with drug problems in Sweden. J Fam Issues.

[19] Richert T, Johnson B, Svensson B (2018). Being a parent to an adult child with drug problems: negative impacts on life situation, health, and emotions. J Fam Issues.

[20] Orford J (2017). How does the common core to the harm experienced by affected family members vary by relationship, social and cultural factors?. Drugs: Education, Prevention and Policy.

[21] Liahaugen Flensburg O, Johnson B, Nordgren J, Richert T, Svensson B (2021). “Something wasn’t right”—parents of children with drug problems looking back at how the troubles first began. Drugs: Education, Prevention and Policy.

[22] Orford J, Velleman R, Copello A, Templeton L, Ibanga A (2010). The experiences of affected family members: a summary of two decades of qualitative research. Drugs: Education, Prevention and Policy.

[23] Liahaugen Flensburg O, Richert T, Väfors Fritz M. Parents of adult children with drug addiction dealing with shame and courtesy stigma. Drugs Educ Prev Policy. 2022;1–10.

[24] McCann TV, Lubman DI (2018). Stigma experience of families supporting an adult member with substance misuse. Int J Ment Health Nurs.

[25] Smith JM, Estefan A (2014). Families parenting adolescents with substance abuse–recovering the mother's voice: a narrative literature review. J Fam Nurs.

[26] Copello A, Templeton L, Orford J, Velleman R (2010). The 5-step method: evidence of gains for affected family members. Drugs: Education, Prevention and Policy.

[27] Orford J, Copello A, Velleman R, Templeton L (2010). Family members affected by a close relative's addiction: the stress-strain-coping-support model. Drugs: Education, Prevention and Policy.

[28] Hogue A, Becker SJ, Wenzel K, Henderson CE, Bobek M, Levy S, Fishman M (2021). Family involvement in treatment and recovery for substance use disorders among transition-age youth: research bedrocks and opportunities. J Subst Abuse Treat.

[29] Miller W, Meyers RM, Tonigan SJ (1999). Engaging the unmotivated in treatment for alcohol problems- a comparison of three intervention strategies. Journalof consulting and clinical psychology.

[30] Smith JE, Meyers RJ (2004). Motivating substance abusers to enter treatment: working with family members.

[31] Meyers RJ, Miller WR, Hill DE, Tonigan JS (1998). Community reinforcement and family training (CRAFT): engaging unmotivated drug users in treatment. J Subst Abuse.

[32] Bischof G, Iwen J, Freyer-Adam J, Rumpf HJ (2016). Efficacy of the community reinforcement and family training for concerned significant others of treatment-refusing individuals with alcohol dependence: a randomized controlled trial. Drug Alcohol Depend.

[33] Archer M, Harwood H, Stevelink S, Rafferty L, Greenberg N (2020). Community reinforcement and family training and rates of treatment entry: a systematic review. Addiction.

[34] Sisson RW, Azrin NH (1986). Family-member involvement to initiate and promote treatment of problem drinkers. J Behav Ther Exp Psychiatry.

[35] Eék N, Romberg K, Siljeholm O, Johansson M, Andreasson S, Lundgren T, Fahlke C, Ingesson S, Bäckman L, Hammarberg A (2020). Efficacy of an internet-based community reinforcement and family training program to increase treatment engagement for AUD and to improve psychiatric health for CSOs: a randomized controlled trial. Alcohol Alcohol.

[36] Hellum R, Bilberg R, Andersen K, Bischof G, Hesse M, Nielsen AS (2022). Primary outcome from a cluster-randomized trial of three formats for delivering Community Reinforcement and Family Training (CRAFT) to the significant others of problem drinkers. BMC Public Health.

[37] Roozen HG, de Waart R, van der Kroft P (2010). Community reinforcement and family training: an effective option to engage treatment-resistant substance-abusing individuals in treatment. Addiction.

[38] Merkouris SS, Rodda SN, Dowling NA (2022). Affected other interventions: a systematic review and meta-analysis across addictions. Addiction.

[39] Waldron HB, Kern-Jones S, Turner CW, Peterson TR, Ozechowski TJ (2007). Engaging resistant adolescents in drug abuse treatment. J Subst Abuse Treat.

[40] Bisetto Pons D, González Barrón R, Botella Guijarro Á (2016). Family-based intervention program for parents of substance-abusing youth and adolescents. J Addict.

[41] Hellum R, Bilberg R, Bischof G, Nielsen AS (2021). How concerned significant others experience Community Reinforcement and Family Training (CRAFT) – a qualitative study. BMC Family Practice.

[42] Osilla KC, Pedersen ER, Tolpadi A, Howard SS, Phillips JL, Gore KL (2018). The feasibility of a web intervention for military and veteran spouses concerned about their partner's alcohol misuse. J Behav Heal Serv Res.

[43] Siljeholm O, Ekström V (2023). A shift in focus: mothers’ descriptions of sharing a child with a co-parent with unhealthy alcohol use after participating in a support program. Addiction Science, Clinical Practice.

[44] Siljeholm O, Edvardsson K, Bergström M, Hammarberg A (2024). Community reinforcement and family training versus counselling for parents of treatment-refusing young adults with hazardous substance use: a randomized controlled trial. Addiction.

[45] Tong A, Sainsbury P, Craig J (2007). Consolidated criteria for reporting qualitative research (COREQ): a 32-item checklist for interviews and focus groups. Int J Qual Health Care.

[46] Braun V, Clarke V (2006). Using thematic analysis in psychology. Research in Psychology.

[47] World Medical Association Declaration of Helsinki. JAMA. 2013;310(20):2191. 10.1001/jama.2013.281053 24141714

[48] McCann TV, Lubman DI (2018). Help-seeking barriers and facilitators for affected family members of a relative with alcohol and other drug misuse: a qualitative study. J Subst Abuse Treat.

[49] Hellum R, Bilberg R, Nielsen AS (2021). He is lovely and awful": The challenges of being close to an individual with alcohol problems. Nordisk Alkohol narkotikatidskrift.

[50] Kirby KC, Benishek LA, Kerwin ME, Dugosh KL, Carpenedo CM, Bresani E, Haugh JA, Washio Y, Meyers RJ (2017). Analyzing components of Community Reinforcement and Family Training (CRAFT): Is treatment entry training sufficient?. Psychol Addict Behav.

[51] Choate P (2015). Adolescent alcoholism and drug addiction: the experience of parents. Behav Sci.

[52] Arnett JJ (2000). Emerging adulthood. A theory of development from the late teens through the twenties. Am Psychol.

[53] Beckwith M, Best D, Savic M, Haslam C, RamezBathish GD, Mackenzie J, Staiger PK, Lubman DI (2019). Social Identity Mapping in Addiction Recovery (SIM-AR): extension and application of a visual method. Addiction Research and Theory.

[54] Dingle GA, Cruwys T, Frings D (2015). Social identities as pathways into and out of addiction. Frontiers in psychology.

[55] LeBouef S, Dworkin J (2023). Digital communication, family identity, and markers of emerging adulthood. J Media Psychol.

[56] Suffoletto B, Pacella M, Huber J, Chung T (2023). Effectiveness of text message interventions with different behavior change techniques on alcohol consumption among young adults: a five-arm randomized controlled trial. Addiction.

[57] Koepke S, Denissen JJA (2012). Dynamics of identity development and separation–individuation in parent–child relationships during adolescence and emerging adulthood – a conceptual integration. Dev Rev.

[58] Guba EG, Lincoln YS (1989). Guba, Egon G. and Yvonna S. Lincoln, fourth generation evaluation.

[59] Mays N, Pope C (2000). Assessing quality in qualitative research. BMJ.

[60] Begley CM (1996). Using triangulation in nursing research. J Adv Nurs.

[61] Hennink MM, Kaiser BN, Marconi VC (2017). Code saturation versus meaning saturation: how many interviews are enough?. Qual Health Res.

[62] The Statistical authority (2022). Educational level in Sweden.

