# Supplementary material for: ‘Before, we ended up in conflicts, now we can provide support’—Experiences of Community Reinforcement and Family Training (CRAFT) for parents of young adults with hazardous substance use
Source: BMC Psychiatry. 2024 Jun 21;24:464. doi: 10.1186/s12888-024-05913-x (PMC11191224; doi:10.1186/s12888-024-05913-x)
Supplement: Supplementary file 2 — Supplementary Material 2. [file 12888_2024_5913_MOESM2_ESM.pdf]

Interview guide

1. Tell us about yourself and your family, who you are the parent of.
   a. Why you applied to the program.
   b. Why then?
   c. Had something special happened?
   d. What appealed to you about this particular study?

2. For how long before applying to the program did you think your son/daughter had an alcohol/drug problem?

3. Have you received any support in your role as a parent in the past regarding your son's/daughter's alcohol or drug problems?

4. What did you hope CRAFT could help you and your youth with when you applied?

5. What did you think of the settings of the sessions? (Individual sessions, online via video)

6. How did you experience the therapist's treatment during the sessions?

7. How did you experience the therapist's knowledge in the field?

8. Which parts of the program did you find most helpful? (See list of themes at the bottom if necessary)

9. What parts of the program did you find least helpful?

10. How has the program affected your understanding of your youth's alcohol/drug use?

11. In what way has the program affected your son's/daughter's use of alcohol/drugs?

12. Alternatives for questions depending on whether the parent indicated that the youth sought treatment or not:

   a. You have indicated that your son/daughter has sought treatment during the time you participated in the study, is that correct?
   b. Was there any particular part of the program that you found particularly helpful and that led to treatment seeking?

Follow-up question: What is your impression of the teratment your son/daughter received? Was it helpful? Was it carried out according to plan?

12. Your son/daughter has not sought treatment during the six months that you participated in the study.
   a. What do you think could lead to him/her seeking care?
   b. If you think back on the CRAFT program, is there something that you missed in it that you think could have increased the chances of leading to treatment seeking.
   c. How do you think the program could develop?

13. How would you describe the relationship with your son/daughter today?
And how has it possibly changed since you applied to the program?

14. Do you feel in need of additional support today?
If yes, what kind of support would you like?

15. Would you recommend other parents in a similar situation to apply to the CRAFT program?

Themes included in CRAFT
1. Introduction and motivational enhancement
2. Functional analysis of young adult’s substance use
3. Positive communication skills
4. Encouraging sobriety and positive reinforcement
5. Parent’s well-being
6. Managing young adult’s substance use
7. Problem-solving and treatment engagement
8. Summary and maintenance of res
